# Assessing data completeness in the international society for the study of pleura and peritoneum (ISSPP) PIPAC database: a multicenter evaluation from 2020–2024

**DOI:** 10.1515/pp-2025-0014

**Published:** 2025-06-30

**Authors:** Magnus Skov Jørgensen, Pernille Shjødt Hansen, Claus Wilki Fristrup, Martin Hübner, Jimmy So, Anne-Cecile Ezanno, Peter Hewett, Miguel Ruiz-Marin, Günther Rezniczek, Özgül Düzgün, Marc Pocard, Francesco Casella, Laura Lay, Marisa Aral, Tarkan Jäger, Felix Laminger, Oliver Glehen, Claire-Angéline Goutard, Laurent Villeneuve, Andrea Di Giorgio, Michael Bau Mortensen

**Affiliations:** Odense PIPAC Center (OPC) and Odense Pancreas Center (OPAC), 11286 Odense University Hospital, Odense, Denmark; Department of Surgery, Odense University Hospital, Odense, Denmark; Lausanne University Hospital CHUV, University of Lausanne, Lausanne, Switzerland; Surgery, National University Hospital, Singapore, Singapore; Department of Surgery, Hôpital d’Instruction des Armées Begin, Saint-Mande, France; Department of Surgery, The Queen Elizabeth Hospital, Woodville South, SA, Australia; Department of Surgery, Reina Sofia University General Hospital, Murcia, Spain; Obstetrics & Gynecology, Marien Hospital Herne, Klinikum der Ruhr-Universität Bochum, Herne, Germany; Department of Surgical Oncology, İstanbul Ümraniye Training and Research Hospital, Istanbul, Türkiye; Department of Digestive, Hepatobiliary Surgery and Liver Transplantation, Pitié-Salpêtrière Hospital, Assistance Publique-Hôpitaux de Paris, Paris, France; Université Paris Cité, CNRS UMR 8175, Inserm UMR-S 1334, NABI Paris, France; General and Upper GI Surgery, University of Verona, Verona, Veneto, Italy; Department of Gynecology Surgical Area at the Institute of Oncology A. H. Roffo, University of Buenos Aires, Buenos Aires, Argentina; General Surgery Department, Centro Hospitalar Universitário de São João, Porto, Portugal; Department of Surgery, Paracelsus Medical University, Salzburg, Austria; Department of Surgery, Center for Peritoneal Carcinomatosis, Hanusch-Krankenhaus, Vienna, Austria; Service de Chirurgie Digestive et Oncologique, Hôpital Lyon Sud, Hospices Civils de Lyon, Lyon, France; Surgical Unit of Peritoneum and Retroperitoneum, Fondazione Policlinico Universitario A. Gemelli IRCCS, Rome, Italy

**Keywords:** database, incomplete data, ISSPP, missing data, PIPAC, peritoneal metastasis

## Abstract

**Objectives:**

In 2020, Pressurized Intraperitoneal Aerosol Chemotherapy (PIPAC) reached stage 2b of the IDEAL framework and a prospective international PIPAC database was launched in June 2020 by the International Society for the Study of the Pleura and Peritoneum (ISSPP). The ISSPP PIPAC database consists of six key elements, which are reported in an annual report. The ISSPP Registry Group decided to investigate data completeness within the ISSPP PIPAC Database.

**Methods:**

Retrospective analysis of data completeness in the six key elements was performed between October 1st and 14th, 2024. This was complemented by an in-depth analysis of missing data in *Response Evaluation, Complications,* and *Follow-up*.

**Results:**

Thirty centers, 950 patients, and 2777 PIPAC procedures were registered in the ISSPP database by October 2024. Sixteen of the 30 centers had included patients. Incomplete data were observed in four of the six key elements. Most centers (7/16) had incomplete data in *Complications*, followed by *Response evaluation* (5/16), and *Follow-up* (2/16). In depth analysis showed that, e.g., for complications, the date and type of the complication was registered in 88 and 89 %, respectively. Incomplete data in *Response evaluation* occurred mainly in the small group of patients evaluated by nonperitoneal regression grading score (non-PRGS, n=316), where no scoring was provided in 211 patients (72 %). *Follow-up* data, such as date of death or reasons for stopping PIPAC, were provided for 86 and 85 % of patients.

**Conclusions:**

Overall data completeness of the ISSPP PIPAC Database was considered satisfactory at the present state, and the ISSPP Registry Group has launched several initiatives to further improve data completeness and quality, to provide solid data sets for future annual reports and other research.

## Introduction

Peritoneal metastasis (PM) is a common condition in patients with gastrointestinal and gynecological cancer. Systemic chemotherapy tends to have a relatively short effect in these patients, reflected in their poor prognosis [1], 2]. A decade ago, Pressurized Intraperitoneal Aerosol Chemotherapy (PIPAC) was introduced to overcome the limitations of systemic chemotherapy during palliative treatment in patients with PM [3]. PIPAC reached stage 2b of the IDEAL framework in 2020, and a prospective international PIPAC database was launched by the International Society for the Study of the Pleura and Peritoneum (ISSPP) and is hosted by Odense Patient data Explorative Network (OPEN) [4], 5]. The ISSPP PIPAC database (PIPAC database) has been implemented using REDCap (Research Electronic Data Capture) [6], a web-based software solution for easy online access, and consists of six key elements: *Patient, Consent, Treatment, Complications, Response evaluation,* and *Follow-up*. The results from the PIPAC database have been described in two previously published annual PIPAC reports [4], 7], and the PIPAC database is considered essential for monitoring indications, complications, and potential clinical effect, while awaiting data from randomized controlled trials.

However, most international databases face challenges during initial construction (e.g., mode of access, implementation of an easy-to-use interface, costs) and continued operation (e.g., maintenance and monitoring costs), as well as data quality and validity (e.g., inaccurate, incomplete, or missing data). The latter may have a negative influence on the final data analyses and conclusions [8], 9]. In 2023, the second annual PIPAC database report showed missing and incomplete data on specific topics like date of death, reasons for stopping PIPAC, and nonperitoneal regression grading score (non-PRGS) response evaluation [7].

As continuous monitoring of missing and incomplete data is an important part of qualifying and improving the database reports, the ISSPP Registry Group decided to evaluate this before publication of the third annual PIPAC database report. Thus, the primary study aim was to assess data completeness within the ISSPP PIPAC database. Secondary objectives included identifying patterns of missing data and proposing improved strategies for data entry, monitoring, and reporting.

## Methods

A retrospective analysis of data completeness within the ISSPP PIPAC database was performed on data exported on 14th of October 2024, which included all data entered since the official launch in June 2020. The primary outcome was to investigate the number of reporting centers with incomplete data in the six key elements. Incomplete data, for this specific purpose, was defined as missing or incorrectly registered data in more than 50 % of the recorded patients within each reporting center. Incorrect data registration was defined as data entered, but not in accordance with the guidelines provided by the ISSPP Registry Group, which are available on the official ISSPP website [10]. Based on this initial data completeness analysis, we evaluated reasons for incomplete data, including potential errors or mistakes owing to the structure and/or presentation of the database (labels, annotations, etc.), and, where relevant or possible, provided potential remedies.

**Figure 1:**
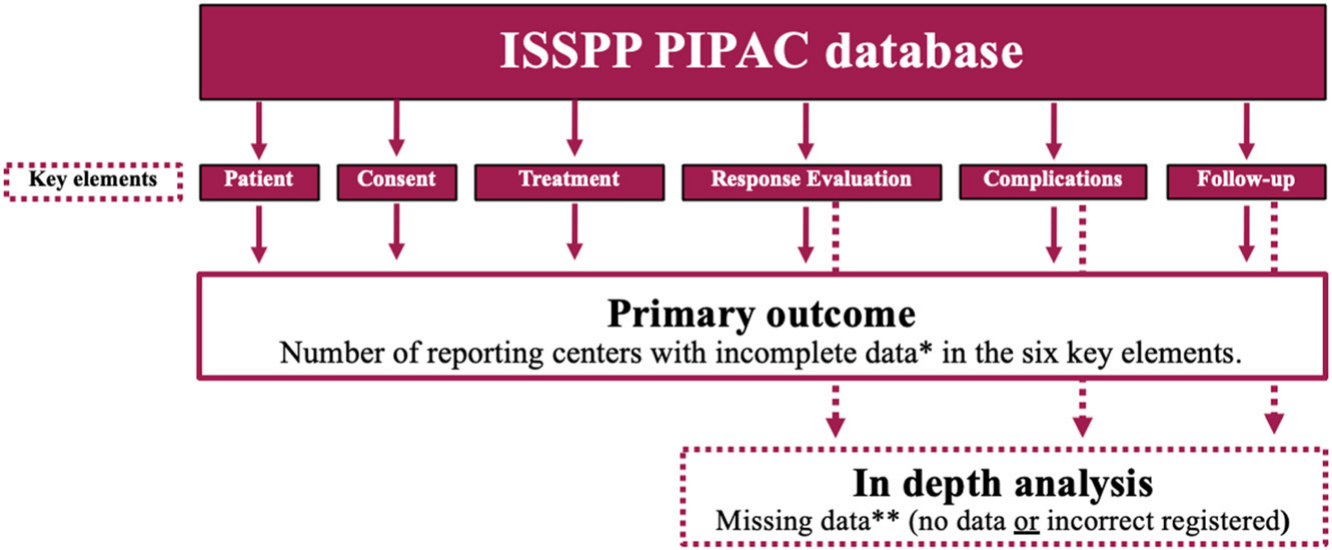
Primary outcome of the study and in-depth analysis of *Response evaluation, Complications,* and Follow-up. *Incomplete data were defined as either missing or incorrectly registered data in more than 50 % of the recorded patients. **Missing data were defined as no or incorrectly registered data.

Further, an in-depth analysis of *Response Evaluation, Complications,* and *Follow-up* were performed to assess the extent of missing data in these three key elements. These elements were chosen due to their significance with regard to reporting PIPAC data, and because these elements had missing data in the second annual PIPAC report [7]. Missing data were defined as no registered data due to either lack of data or incorrectly registered data (Figure 1). The in-depth analysis of complications included an examination of the centers that reported most complications and the frequency of severe complications per PIPAC center.

The governance, legal aspects, ethical framework, variables, and implementation of the ISSPP PIPAC Database have been previously described in detail [4], 7] (Table 1).

**Table 1:** A summary of the ISSPP PIPAC database. The in-depth analysis included the three key elements *Response Evaluation, Complications,* and *Follow-up*.

Name	The ISSPP PIPAC database
Description	Web-based REDCap database solution for easy online access and consisting of six key elements: *Patient, Consent, Treatment, Complications, Response evaluation,* and *Follow-up*
Purpose	To monitor global PIPAC activity, perform quality assessment, and support international PIPAC benchmarking and research
Years of data registration	Since June 2020
Method of data collection	Manual online input by PIPAC centers
No. of annual reports	2
No. of registered centers^a^	30
No. of reporting centers^a^	16
No. of patients^a^	950
No. of PIPAC directed treatments^a^	2,777
In depth analysis	
*Response evaluation*	2,705 histological response evaluations
*Complications*	3,189 complications
*Follow-up*	950 patients

^a^As of October 14th, 2024.

## Results

### Primary outcome

As of October 14th, 2024, the ISSPP PIPAC database contained data from 2,777 PIPAC procedures in 950 patients. Thirty PIPAC centers were registered in the database, and 16 (53 %) centers had actually included patients. Incomplete data were observed in four of the key elements, but not in *Treatment* and *Patient*. Most incomplete data were in *Complications, Response Evaluation*, and *Follow-up* (Table 2). Six of the 16 reporting centers (38 %) had no incomplete data, and no reporting center had incomplete data in all key elements.

**Table 2:** Reporting centers (n=16) with incomplete data^a^ in the six key elements.

Key element	Number of reporting centers with incomplete data, n (%)
Patient	0 (0)
Consent	1 (7)
Treatment	0 (0)
Response evaluation	5 (31)
Complications	7 (44)
Follow-up	2 (13)

^a^Incomplete data were defined as missing or incorrectly registered data in more than 50 % of the recorded patients within each reporting center.

All centers had complete data in *Patient, Consent,* and *Treatment* elements – except one center where the majority of patients did not consent to having their information passed on to the database.

Reasons for incomplete data in *Response Evaluation* were due to missing data or incorrect registration of data, see below (5 centers).

**Figure 2:**
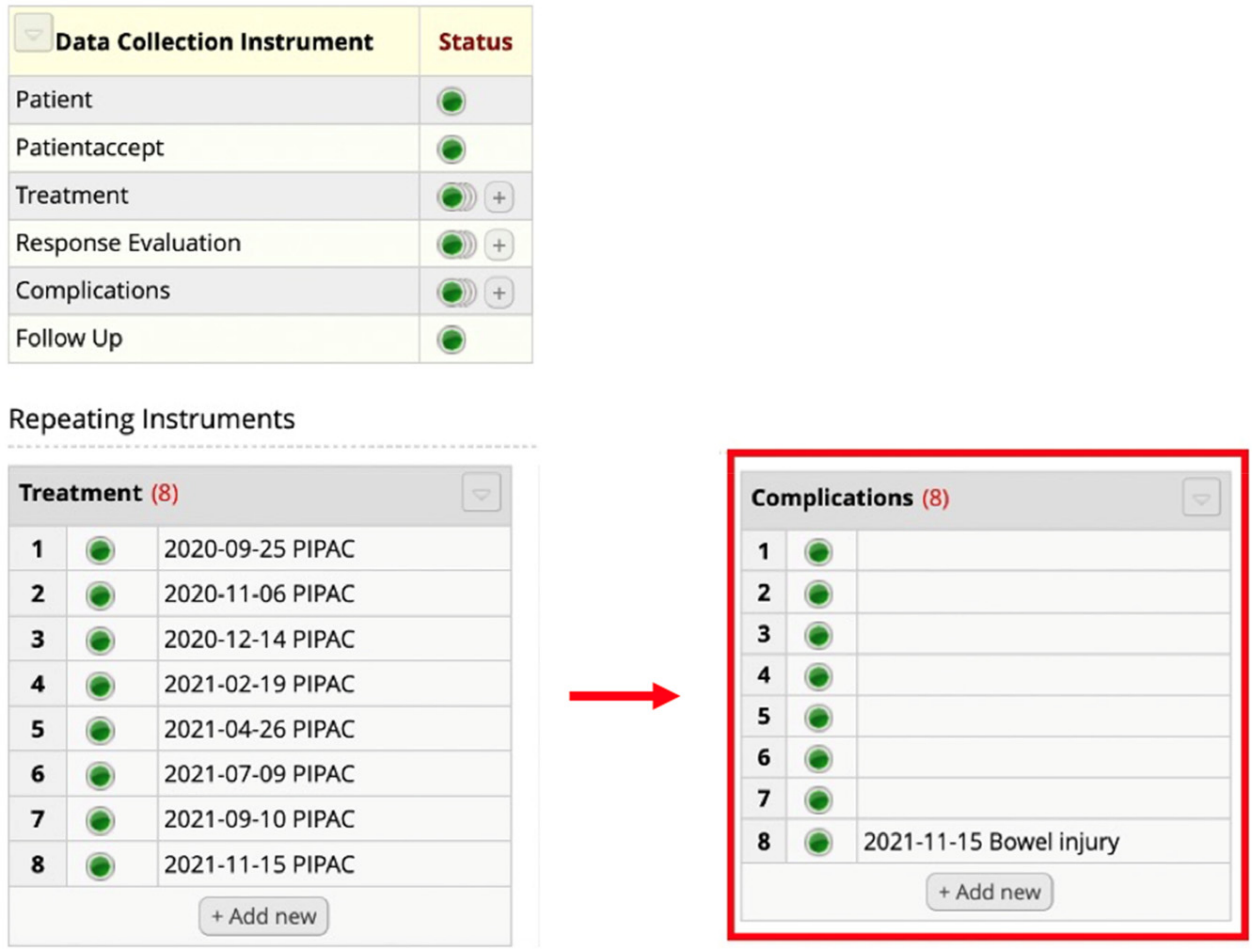
Incompleteness in the key element *Complications*. The patient was treated with eight PIPAC directed treatments. As highlighted in red, eight complications were registered but only one complication was provided with information as to the character of the problem (e.g., bowel injury). Seven of the complications had no registered data and are considered “empty.”

*Complications* had the highest amount of incomplete data (7 centers). This was due to incorrect registration of data. The major cause being centers incorrectly marking new complications without registering further data and thereby creating “empty” complication records1An “empty complication” field is defined as a recorded complication without any additional registered data (e.g., type of complication). (Figure 2). For a more detailed description of this topic, please read below.

In *Follow-up*, incomplete data (no follow-up) were observed in two (13 %) reporting centers, only.

### Results from in-depth analysis

The number of missing data in the key elements *Response Evaluation, Complications*, and *Follow-up* are listed in Table 3.

**Table 3:** In depth analysis of missing data in *Response Evaluation, Complications,* and *Follow-up*. Missing data are divided into no data and incorrect data. No data refers to no data was registered. Incorrect data mean data were registered but not in accordance with the guidelines provided by the ISSPP Registry Group.

Key element	Total data, n (%)	Missing data, n (%)	
		No data	Incorrect data
Response evaluation (n=2,705)			
PRGS	2,077 (75)	39 (1.9)	16 (0.8)
Non-PRGS	293 (11)	211 (72)	1 (0.3)
None	308 (11)	–	26 (8)
Unknown/not specified	27 (3.3)	–	–
Complications (n=3,189)			
Date of complication	3,189 (100)^a^	0 (0)	375 (12)
Type of complication	3,189 (100)	0 (0)	360 (11)
Dindo-Clavien	38 (1.2)	6 (16)	0 (0)
CTCAE	3,151 (99)	472 (15)	0 (0)
Follow-up (n=950)			
Date of follow-up	950 (100)	54 (5.7)	8 (0.8)^b^
Date of death	574 (100)	79 (14)	–
Reasons for stopping PIPAC	950 (100)	143 (15)	0 (0)

^a^One PIPAC, procedure may be accompanied by more than one registered complication, ^b^Observed in two PIPAC, centers. PRGS, peritoneal regression grading score, CTCAE; Common Terminology Criteria for Adverse Events.

*Response evaluation* was registered in 2,705 out of 2,777 procedures (97 %). Evaluation by Peritoneal Regression Grading Score (PRGS)2The Peritoneal Regression Grading Score (PRGS) is a validated objective tool used to evaluate the histological response in peritoneal biopsies obtained during PIPAC-directed treatments. had no or incorrect data in 2.7 %, only. Non-PRGS3Non-PRGS refers to alternative histological tools used to evaluate the microscopic response to treatment. was performed in 293 (11 %) procedures but the option “not available” was chosen in 211(72 %). The option “None” was chosen in 308 procedures (11 %), yet a date-matched biopsy was used for response evaluation and registered in 26 (8 %) of the procedures (i.e., incorrect data).

The complication data were noted in 88 % of all procedures and the type of complication was listed in 89 %. Dindo-Clavien (DC) and Common Terminology Criteria for Adverse Events (CTCAE) classification were missing in 16 and 15 % of the complication reports, respectively. Two PIPAC centers were responsible for 84 % of the total number of registered complications. The two centers performed 68 % of all PIPAC procedures.

Frequency of complications DC≥3b and adverse events CTCAE≥3 per PIPAC varied from 0–3.3 % and 0–5.5 %, respectively, among reporting centers with more than 30 PIPACs performed (Table 4).

**Table 4:** Frequency of Dindo-Clavien ≥ 3b and CTCAE≥3 in PIPAC procedures among centers with more than 30 PIPACs performed.

Center	Dindo-Clavien≥3b, %	CTCAE≥3, %
1	1.6	5.5
2	3.3	0
3	1.9	2.9
4	0	3.8
5	0.8	0.8
6	0	0
7	0.2	4.6
8	0	0

Regarding *Follow-up*, 54 patients (5.7 %) had no data, while incorrect follow-up date occurred in eight patients (0.8 %) due to no follow-up performed in two centers.

Date of death was provided in 86 % of the patients reported as having died. Reasons for stopping PIPAC directed therapy were available in 85 %, but in 15 % of the patients, the reason was “other,” but not specified.

## Discussion

The voluntary, noncommercial ISSPP PIPAC database has included PIPAC centers and patient data for more than 4 years and two annual reports have been published [4], 7]. Currently, 16 centers are reporting their data on PIPAC procedures to the ISSPP PIPAC database as of October 2024. Continuous monitoring of data is relevant for several reasons (e.g., database design, use, variable definitions, and reporting), and assessment of data completeness and accuracy are important criteria for this evaluation. This study assesses data completeness within the ISSPP PIPAC database and represents the first systematic audit of a prospective international PIPAC registry.

Incomplete data, when defined as either missing data in more than 50 % of the included patients or incorrectly registration of data in more than 50 %, were found in four of six key elements of the database. The key elements were *Complications* (7/16 reporting centers), *Response Evaluation* (5/16), *Follow-up* (2/16), and *Consent* (1/16). Data were complete in the key elements *Patient* and *Treatment*.

The incomplete data in *Consent* resulted from one center, where most patients did not consent to having their information registered in the database. Consequently, data from these patients were excluded from this study’s analysis.

Reasons for incomplete data in *Complications* and *Response evaluation* were mainly incorrect registration of data, and the reporting centers often created the same errors. In *Complications*, the main error was registration of complications without providing subsequent data and thereby creating “empty” complication lists. The misunderstanding seems to arise when investigators believe empty complications mean no complication has occurred. Thus, important to emphasize in the available database guidelines, that when pressing “yes” to the variable “complication (yes/no)” under the key element *Treatment*, the investigator must register the new complication separately under the key element *Complication*. If “no” is chosen, then no complications occurred, and no separate complications should be registered.

One error in registering complications have previously been a misuse of the “other”-field, with some centers listing numerous complications in the text field. However, this was not observed in this data overview.

**Figure 3:**
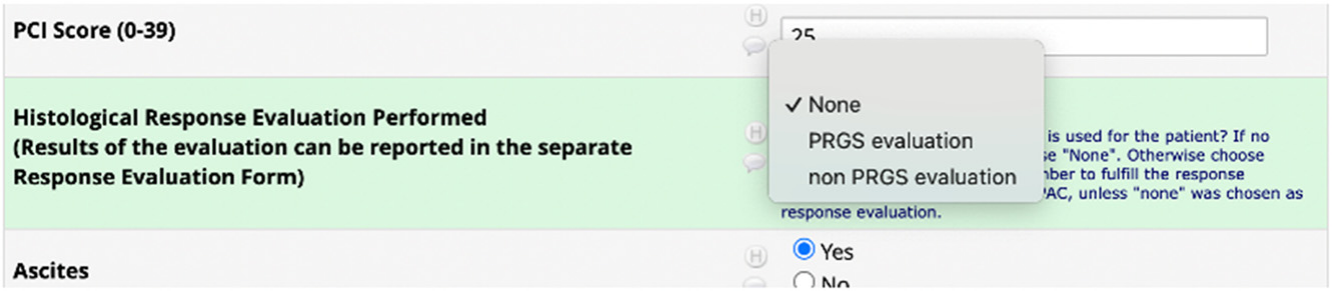
Under the key element treatment, the investigator should choose which kind of histological response evaluations is performed. There are three options: None, PRGS evaluation, or non-PRGS evaluation.

**Figure 4:**
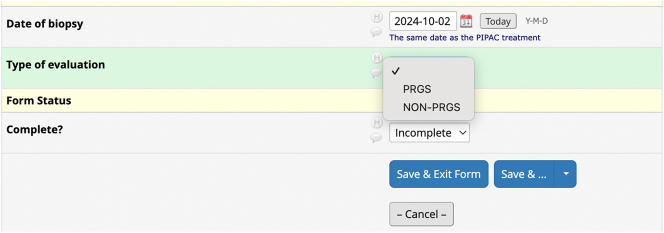
Creation of a new field under the key element Response evaluation. The investigator chooses whether PRGS or non-PRGS was performed and is prompted to provide additional response data.

Regarding *Response evaluation*, the correct method to register data is to select the type of histological response evaluation that was performed during the specific PIPAC directed treatment under the key element Treatment (Figure 3). As underlined in the guideline, the investigator should not create a separate response evaluation, if they choose “none.” On the other hand, if the investigator chooses “PRGS evaluation” or “non PRGS evaluation,” it will be necessary to create a response evaluation (Figure 4).

In the *Patient* element, one center used initials instead of recognizable numbers as patient-ID. Thereby the center risks that two patients are included with the same initials, and therefore it cannot be recommended to use initials as patient-ID. This will be emphasized in an updated database guideline.

The in-depth analysis of *Complications, Response Evaluation,* and *Follow-up* revealed missing data in 72 % of patients with non-PRGS response evaluation. Because of overlapping between the two categories “no data” and “incorrect data,” it is difficult to differentiate whether the high number of missing data is caused only by incorrectly registration or that some investigators forget to register the non-PRGS response. The same argument applies for the missing data in *Complication* and *Follow-up* elements. However, no other variables in the in-depth analysis exceeded 16 % of missing data, which must be considered very satisfactory at the present state. In comparison, 15 out of 315 patients (4.8 %) were excluded due to “incomplete data” in a recent international registry of patients with peritoneal metastases (PM) undergoing cytoreductive surgery and hyperthermic intraperitoneal chemotherapy (HIPEC) [11]. The term “incomplete data” was not further defined. Direct comparison between studies is challenging, since the present study evaluates incomplete data on a variable level, whereas the HIPEC study focused on assessment on patient level. A conservative estimate of patients excluded from the ISSPP PIPAC database due to “incomplete data” would be less than 10 %. On a variable level, one of the major databases including 492,242 patients with invasive breast cancer, the clinical stage information was missing in 29 % of the patients [12]. In that perspective, the current extent of incomplete data in the ISSPP PIPAC database seems acceptable.

It is well known that major multicenter databases must accommodate incomplete data, and that achieving a complete dataset for all included patients is unlikely [8], 13]. Missing data may have quite different clinical implications depending on the topic of the database. One study of missing data in a national health database in the United States concluded that a high prevalence of missing data was associated with differences in overall survival. This emphasizes the importance of complete documentation and registration [8]. Data completeness also depends on the dedication and resources of the involved centers, the hosting center, analysis group, and affiliated clinical societies.

Lack of data completeness is also a well-known problem in both pro- and retrospective PIPAC studies. A systematic review on quality of life in patients treated with PIPAC found moderate risk of bias due to missing data in six of nine included studies [14]. A narrative review on response evaluation in PIPAC detected a substantial amount of missing data or discontinued treatments. The review showed that missing data or discontinued treatments in more than half of patients occurred in 62 % of the included studies [15]. Similarly, this critical database evaluation detected 31 % of the reporting PIPAC centers having incomplete data in the key element *Response evaluation*. Absence of response evaluations may affect interpretations of PIPAC safety and efficacy, potentially biasing clinical decision-making or outcome reporting.

A retrospective study on safety of PIPAC combined with other surgical procedures reported that only 6 % of the patients have been excluded due to missing data, but this is probably due to selection [16]. Another systematic review on reasons for stopping PIPAC showed that missing data ranged from 0 to 24.4 % among the included studies [17]. These numbers are in line with the present study, where reasons for stopping PIPAC were missing in 15 % of the patients. Furthermore, the systematic review reported that one of the main limitations of the study was missing data [17]. Therefore, continued investments in data collection and correct registration remain a critical step toward more complete and trustworthy real-world data – also in the ISSPP PIPAC Database.

### Limitations

Fourteen centers (47 %) registered in the ISSPP PIPAC database had not included any patients, which may introduce reporting bias. Additionally, no inter-rater reliability checks were performed in this study, potentially leading to inconsistent data interpretation across centers and compromising the accuracy and comparability of recorded outcomes. The absence of external audits may allow errors, inconsistencies, or deviations from data entry protocols to go unnoticed, thereby reducing the overall reliability and integrity of the database.

Additionally, some centers may delay data entry into the ISSPP PIPAC database, increasing the risk of missing information. To minimize this, centers are encouraged to register data in real time, while adhering to the detailed instructions provided.

### New database initiatives by the ISSPP Registry Group

The high number of reporting centers with the same incorrect registration of data may reflect a fundamental error in the structure of the database, making it less intuitive and more difficult to register data correctly. To prevent this, new initiatives have been launched, based on discussions within the ISSPP Registry group during the ISSPP/PSOGI meeting in Lyon, September 2024.

The present study shows that the number of registered complications per PIPAC procedure varied across the reporting centers. Low grades of CTCAE and DC are considered less important and recent studies have only reported CTCAE≥3 or DC≥3b [1], 17], 18]. Therefore, and to reduce workload during data registration, it was decided that only CTCAE≥3 will be registered in the database in the future. However, all surgical complications will be registered using the Dindo-Clavien classification.

To minimize incomplete data in the future, Odense PIPAC Center and the ISSPP Registry Group launched an improved and individual PIPAC support program for active and new centers in 2024. An online email help desk has been established (ouh.a.pipac@rsyd.dk), which will guide both new and already reporting PIPAC centers. Also, a support team with two technical assistants has been made available by Odense PIPAC Center. Their help includes email reminders to each of the reporting PIPAC centers in need of corrections to their registered data. A new set of recommendations for registering data in the ISSPP PIPAC database has been sent to all centers, and this detailed guide is available on the ISSPP webpage as well. Furthermore, helpful text-fields are now inserted below the most difficult variables in the database to ensure more consistent data. Finally, the ISSPP Registry Group decided on a simpler approach to data from the ISSPP PIPAC Database.

## Conclusions

International databases must deal with missing and inaccurate data, and this was confirmed in four of the six key elements within the ISSPP PIPAC Database. However, overall data completeness was considered satisfactory at the present state, and the ISSPP Registry Group has launched several initiatives to improve data completeness and quality to enrich future data sets and annual reports.

## References

[1] Di Giorgio A, Macrì A, Ferracci F, Robella M, Visaloco M, De Manzoni G (2023). 10 Years of pressurized intraperitoneal aerosol chemotherapy (PIPAC): a systematic review and Meta-analysis. Cancers (Basel).

[2] Alyami M, Hübner M, Grass F, Bakrin N, Villeneuve L, Laplace N (2019). Pressurised intraperitoneal aerosol chemotherapy: rationale, evidence, and potential indications. The Lancet Oncol.

[3] Solass W, Kerb R, Mürdter T, Giger-Pabst U, Strumberg D, Tempfer C (2014). Intraperitoneal chemotherapy of peritoneal carcinomatosis using pressurized aerosol as an alternative to liquid solution: first evidence for efficacy. Ann Surg Oncol.

[4] Mortensen MB, Glehen O, Horvath P, Hübner M, Hyung-Ho K, Königsrainer A (2021). The ISSPP PIPAC database: design, process, access, and first interim analysis. Pleura Perit.

[5] Baggaley AE, Lafaurie G, Tate SJ, Boshier PR, Case A, Prosser S (2022). Pressurized intraperitoneal aerosol chemotherapy (PIPAC): updated systematic review using the IDEAL framework. Br J Surg.

[6] Harris PA, Taylor R, Thielke R, Payne J, Gonzalez N, Conde JG (2009). Research electronic data capture (REDCap)–a metadata-driven methodology and workflow process for providing translational research informatics support. J Biomed Inform.

[7] Mortensen MB, Casella F, Düzgün Ö, Glehen O, Hewett P, Hübner M (2023). Second annual report from the ISSPP PIPAC database. Pleura Perit.

[8] Yang DX, Khera R, Miccio JA, Jairam V, Chang E, Yu JB (2021). Prevalence of missing data in the national cancer database and association with overall survival. JAMA Netw Open.

[9] Rabe BA, Day S, Fiero MH, Bell ML (2018). Missing data handling in non-inferiority and equivalence trials: a systematic review. Pharm Stat.

[10] ..

[11] Arjona-Sanchez A, Aziz O, Passot G, Salti G, Serrano A, Esquivel J (2023). Laparoscopic cytoreductive surgery and hyperthermic intraperitoneal chemotherapy: long term oncologic outcomes from the international PSOGI registry. Eur J Surg Oncol.

[12] Hoskin TL, Boughey JC, Day CN, Habermann EB (2019). Lessons learned regarding missing clinical stage in the national cancer database. Ann Surg Oncol.

[13] Hunt NB, Gardarsdottir H, Bazelier MT, Klungel OH, Pajouheshnia R (2021). A systematic review of how missing data are handled and reported in multi-database pharmacoepidemiologic studies. Pharmacoepidemiol Drug Saf.

[14] Li Z, Wong LCK, Sultana R, Lim HJ, Tan JW, Tan QX (2022). A systematic review on quality of life (QoL) of patients with peritoneal metastasis (PM) who underwent pressurized intraperitoneal aerosol chemotherapy (PIPAC). Pleura Perit.

[15] Roensholdt S, Detlefsen S, Mortensen M, Graversen M (2023). Response evaluation in patients with peritoneal metastasis treated with pressurized IntraPeritoneal aerosol chemotherapy (PIPAC). J Clin Med.

[16] Robella M, Hubner M, Sgarbura O, Reymond M, Khomiakov V, di Giorgio A (2022). Feasibility and safety of PIPAC combined with additional surgical procedures: PLUS study. Eur J Surg Oncol.

[17] Ezanno AC, Malgras B, Pocard M (2023). Pressurized intraperitoneal aerosol chemotherapy, reasons for interrupting treatment: a systematic review of the literature. Pleura Perit.

[18] Daniel SK, Sun BJ, Lee B (2023). PIPAC for gastrointestinal malignancies. J Clin Med.

